# Human papillomavirus prevalence and type-distribution among women in Zhejiang Province, Southeast China: a cross-sectional study

**DOI:** 10.1186/s12879-014-0708-8

**Published:** 2014-12-19

**Authors:** Xiao-Xiang Liu, Xing-Li Fan, Yue-Ping Yu, Lei Ji, Jie Yan, Ai-Hua Sun

**Affiliations:** Faculty of Basic Medicine, Zhejiang Medical College, Hangzhou, 310053 Zhejiang P.R. China; Division of Basic Medical Microbiology, State Key Laboratory for Diagnosis and Treatment of Infectious Diseases, First Affiliated Hospital of Zhejiang University, Hangzhou, 310003 Zhejiang P.R. China; Department of Medical Microbiology and Parasitology, College of Medicine, Zhejiang University, Hangzhou, 310058 Zhejiang P.R. China

**Keywords:** Human papillomavirus (HPV), Prevalence, Genotyping, Cytology

## Abstract

**Background:**

Human papillomavirus (HPV) infection is the main etiological factor for cervical cancer and premalignant lesions of the cervix. The purposes of the present study were to determine the prevalence of type-specific HPV infections and the association of different HPV types with cervical dysplasia among women in Zhejiang province, Southeast China.

**Methods:**

A total of 15,267 women presenting to a gynaecological outpatient clinic were enrolled in this study. Women were screened for HPV in addition to routine cervical cytology testing. Microarray hybridization and liquid-based cytology tests were used to detect HPV genotypes and cervical cytology, respectively.

**Results:**

Based on the population attending a gynaecological outpatient clinic, overall prevalence of any 23 HPV type was 22.8% and multiple HPV infection was found in 4.0% of all the outpatients. HPV prevalence showed bimodal age distribution, with a peak (55.7%) at the ≤20 age group and a second one (35.5%) at >60 age group. In total samples, the five most frequent types were HPV 16 (4.4%), 58 (2.9%), 52 (2.7%), 33 (2.2%) and 11 (1.9%). Overall HPV prevalence increased with the severity of the cytologic result. Analysis through crude odds ratios (ORs) revealed that the cervical lesion risk of HPV-infected women increased to about 26-fold of uninfected women (OR 26.1, 95% CI 22.4 to 30.3). The five most risky HPV types associated with abnormal cytology were HPV 73, 16, 82, 45 and 51.

**Conclusions:**

This study provided baseline data on HPV prevalence in women attending a gynecological outpatient clinic in Zhejiang province. Our data will supply guidance for the primary screening and vaccination program for cervical cancer in this area.

**Electronic supplementary material:**

The online version of this article (doi:10.1186/s12879-014-0708-8) contains supplementary material, which is available to authorized users.

## Background

Cervical cancer is the second most common cancer among women, with an estimated 530,000 new cases and 275,000 deaths occurring each year in the world [1],[2]. There are an estimated 88% of the annual incidences occurring in developing countries, such as China, where approximately 75,500 new cases and 34,000 deaths occur every year [2],[3]. According to epidemiological and molecular studies, it is well-established that human papillomavirus (HPV) infection is the main etiological factor for cervical cancer and premalignant lesions of the cervix [4],[5]. So far, over 100 different HPV genotypes have been identified, of which nearly 40 types are associated to lesions of the female genital tract and are classified as low-risk (LR) or high-risk (HR) based on their potential to cause cancer [5]. There are at least 18 HR-HPV types [6],[7], of which HPV 16, 18, 31, 33, 35, 45, 52, and 58 contribute to 91% of the invasive cervical cancers diagnosed and HPV 16 and 18 account for approximately 70% of all the case worldwide each year [8], while HPV 6, 11, 42, 43 and 44 are classified as “LR types”, which are associated with hyperplastic lesions such as genital warts [5].

Since persistent infection of HPV is a necessary cause of cervical cancer worldwide, DNA tests for the detection of HPV can be used as a primary screening method for precancerous lesions and cervical cancer, and for triage of women with atypical or borderline cervical smears [9],[10]. In addition, the data of HPV type-specific distribution will provide guidance for the vaccination program for cervical cancer. Prophylactic vaccines against HPV have been developed and approved in more than 100 countries around the world [11]. However, in China, HPV vaccines are still under clinical trials for government approval. There are two available HPV vaccines, a bivalent vaccine targeted at HPV 16 and 18 and a quadrivalent HPV vaccine targeted at HPV 6, 11, 16 and 18 [12],[13]. These current preventive HPV vaccines offer protection only against a few HPV genotypes. HPV prevalence and subtype distribution varied greatly amongst different geographic areas [14],[15]. Since limited cross-protection was present between HPV types [16], knowledge of geographical differences in HPV type-specific distribution might be very valuable for predicting the effect of current prophylactic vaccines and forming the basis for the second generation vaccines targeted to specific regions.

To the best of our knowledge, limited large scale epidemiologic data of HPV infection have been reported in Zhejiang Province, a coastal region in Southeast China. In this study, we collected 15,267 samples from women attending regular gynecological visits in Zhejiang Province. All women were offered HPV test and ThinPrep cytology test (TCT) to analyze the association between HPV genotype and cervical cytology. The overall, age-specific, genotype-specific, and state-specific prevalence of HPV in Zhejiang province will provide guidance for the future screening strategies of cervical cancer and vaccination program.

## Methods

### Study population

The study population consisted of 15,267 women (age, 18–79 years; mean, 38.3 ± 7.1 years) attending a gynecological outpatient clinic between January 2010 to December 2013 in Zhejiang Province. A woman was considered eligible to enter the study if she a) had current or past sexual activity, b) was not pregnant at the time of enrollment, c) had never been screened or treated for cervical cancer, d) had not undergone a total uterus or cervix resection, e) agreed to undergo an HPV test and a TCT, and f) agreed to participate in the present study. This research was conducted in accordance with the Declaration of Helsinki and a protocol approved by the Ethics Committee of Zhejiang Medical College (Hangzhou, China).

### TCT and pathological diagnosis

Exfoliated cervical cells were collected from the orifice of uterus and endocervical canal with a cervical brush. The cells were washed into vials containing ThinPrep preservative solution and were sent to the cytology laboratory for TCT analysis. Cytological findings were evaluated according to the Bethesda classification system [17] and were classified as follows: a) negative; b) atypical squamous cells of undetermined significance (ASC-US); c) low-grade squamous intraepithelial lesion (LSIL); d) atypical squamous cells that cannot exclude HSIL (ASC-H); e) high-grade squamous intraepithelial lesion (HSIL).

### HPV detection and genotyping

HPV DNA was extracted from the cytological remnants, and was detected and genotyped using a commercial HPV Genotyping Kit for 23 HPV types (Yaneng Bioscience (Shenzhen) Co., Ltd, China), including 5 LR types (6, 11, 42, 43 and 44), and 18 HR types (16, 18, 31, 33, 35, 39, 45, 51, 52, 53, 56, 58, 59, 66, 68, 73, 82 and 83). The kit employs DNA amplification to detect HPV positivity and a microarray format with a nylon membrane onto which HPV genotype-specific oligonucleotide probes have been immobilized to simultaneously identify 23 HPV genotypes. The HPV test was conducted according to the manufacturer’s recommendation and was validated through the use of positive and negative controls at each shift.

### Statistical analysis

The data were analyzed using SPSS software for windows (version 16.0). The prevalence of HPV according to age and cytological lesion types was analyzed. The 95% confidence intervals (CI) of HPV prevalence were based on normal approximations. Chi-square test was used to compare the proportions between different groups, with crude odds ratios (ORs) and 95% CI calculation to estimate the risk of each HPV type for cervical lesions. Linear-by-linear association test was used to investigate trend in distribution of HPV infection according to grade of cytology abnormalities. *P*-values were two-sided, and statistical significance was defined as p < 0.05.

## Results

### Overall HPV prevalence

A total of 15,267 female out patients were enrolled in this study, of which 3,486 cases (22.8%) were positive for HPV infection. The prevalence of single HR-HPV was 15.8% (2408/15267) and single LR-HPV was 3.1% (474/15267). Multiple HPV infection was found in 4.0% (604/15267) of all the samples. Among women with multiple infections, 82.0% (495/604) were infected with two HPV types, 12.4% (75/604) were with three types and 5.6% (34/604) were with more than three types. Of the HPV positive cases, 69.1% (2408/3486) were single HR-HPV infections, 13.6% (474/3486) were single LR-HPV infections, and 17.3% (604/3486) were infected by multi-types, respectively.

### HPV prevalence by age

**Figure 1:**
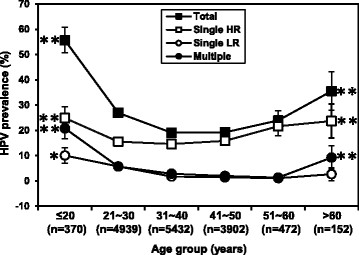
**Age-specific prevalence of total HPV, single HR-HPV, single LR-HPV and multiple HPV with 95% CI.** Significant differences between the peak and lowest prevalence are indicated as: *p < 0.05; **p < 0.01.

HPV prevalence by age group was examined in order to assess age trends in relation to HPV infection in more detail. Women were 18–79 years old (mean age 38.3) and were divided into six groups according to their age. As shown in Figure 1, the age-specific prevalence of total HPV infection exhibited a peak of 55.7% (206/370, Pearson’s Chi Square test, x^2^ = 276.3, p < 0.001) at ≤20 years old and a second peak 35.5% (54/152, Pearson’s Chi Square test, x^2^ = 25.557, p < 0.001) at the age group > 60 years old. Similarly, the prevalence of single HR-HPV and multiple HPV also exhibited the bimodal age distribution. However, single LR-HPV infection showed relatively flat curve.

### HPV prevalence by type

**Figure 2:**
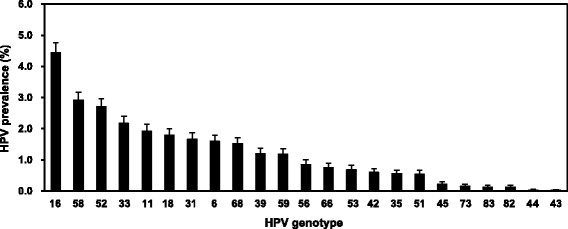
**Overall prevalence of different HPV genotypes.** There are 18 HR-HPVs (16, 18, 31, 33, 35, 39, 45, 51, 52, 53, 56, 58, 59, 66, 68, 73, 82 and 83) and 5 LR-HPVs (6, 11, 42, 43, and 44).

Using the microarray method, totally 23 HPV types were detected (18 HR types, 5 LR types). Overall, HPV 16 was the most commonly detected genotype present with a prevalence of 4.4% (678/15267), followed by HPV 58 (2.9%, 444/15267), HPV 52 (2.7%, 414/15267), HPV 33 (2.2%, 331/15267) and HPV 11 (1.9%, 294/15267) (Figure 2). Although HPV 11 is a LR type, its prevalence is higher than some other HR-HPVs, such as HPV 18 and HPV 31.

### HPV prevalence by cervical cytology

As shown in Table 1, the overall HPV prevalence in normal, ASC-US, LSIL and HSIL cytology samples were 16.8% (2330/13891), 80.2% (644/828), 91.2% (237/260) and 94.4% (102/108), respectively. As expected, HPV prevalence increased with the severity of the cytologic result (Pearson’s Chi Square test, p < 0.001; Linear-by-Linear association test, p < 0.001). Moreover, multiple HPV positivity also exerted a similar increasing trend with the development of cytology abnormalities (Pearson’s Chi Square test, p < 0.001; Linear-by-Linear association test, p < 0.001).

**Table 1:** **HPV type distribution by cytological results**

HPV type	Normal (13891)	ASC-US (828)	LSIL (260)	ASC-H (180)	HSIL (108)
	N (%)	N (%)	N (%)	N (%)	N (%)
HR-HPV					
16	345 (2.5)	180 (21.7)	65 (25.0)	65 (36.1)	23 (21.3)
18	154 (1.1)	89 (10.7)	18 (6.9)	9 (5.0)	3 (2.8)
31	141 (1.0)	75 (9.1)	22 (8.5)	5 (2.8)	11 (10.2)
33	190 (1.4)	63 (7.6)	26 (10.0)	25 (13.9)	27 (25.0)
35	67 (0.5)	12 (1.4)	4 (1.5)	2 (1.1)	0 (0.0)
39	140 (1.0)	34 (4.1)	5 (1.9)	4 (2.2)	1 (0.9)
45	17 (0.1)	11 (1.3)	2 (0.8)	2 (1.1)	2 (1.9)
51	42 (0.3)	19 (2.3)	13 (5.0)	4 (2.2)	5 (4.6)
52	270 (1.9)	90 (10.9)	38 (14.6)	12 (6.7)	4 (3.7)
53	87 (0.6)	10 (1.2)	5 (1.9)	3 (1.7)	0 (0.0)
56	92 (0.7)	9 (1.1)	22 (8.5)	5 (2.8)	2 (1.9)
58	264 (1.9)	93 (11.2)	32 (12.3)	12 (6.7)	43 (39.8)
59	154 (1.1)	21 (2.5)	1 (0.4)	3 (1.7)	2 (1.9)
66	70 (0.5)	29 (3.5)	9 (3.5)	6 (3.3)	1 (0.9)
68	186 (1.3)	17 (2.1)	17 (6.5)	7 (3.9)	4 (3.7)
73	8 (0.1)	4 (0.5)	7 (2.7)	4 (2.2)	1 (0.9)
82	9 (0.1)	3 (0.4)	3 (1.2)	4 (2.2)	0 (0.0)
83	15 (0.1)	3 (0.4)	0 (0.0)	1 (0.6)	0 (0.0)
LR-HPV					
6	221 (1.6)	15 (1.8)	4 (1.5)	2 (1.1)	1 (0.9)
11	257 (1.9)	24 (2.9)	8 (3.1)	5 (2.8)	0 (0.0)
42	73 (0.5)	13 (1.6)	3 (1.2)	2 (1.1)	0 (0.0)
43	2 (0.0)	1 (0.1)	0 (0.0)	0 (0.0)	0 (0.0)
44	3 (0.0)	0 (0.0)	1 (0.4)	0 (0.0)	0 (0.0)
Any type	2330 (16.8)	664 (80.2)	237 (91.2)	153 (85.0)	102 (94.4)
Single	1921 (13.8)	551 (66.5)	194 (74.6)	133 (73.9)	83 (76.9)
Multiple	409 (2.9)	113 (13.6)	43 (16.5)	20 (11.1)	19 (17.6)
Negative	11561 (83.2)	164 (19.8)	23 (8.8)	27 (15.0)	6 (5.6)

**Figure 3:**
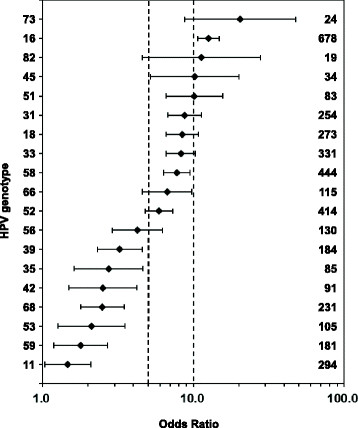
**Risk of each HPV type for cervical lesions.** Crude odds ratios with 95% confidence intervals for the prevalence of HPV associated with abnormal cytology (ASC-US, LSIL, ASC-H and HSIL) are provided. Numbers on right margin are the total number of positive specimens infected by the specified type. The prevalence of HR-HPV 83, LR-HPV 6, 43 and 44 in abnormal specimens showed no significant difference to normal specimens (Pearson’s Chi Square test, p > 0.05) and no ORs were provided. Reference lines for ORs of 5 and 10 are added for readability.

In order to estimate the risk of HPV infection to have abnormal cytology, the crude ORs for the prevalence of HPV associated with abnormal cytology (ASC-US, LSIL, ASC-H and HSIL) are shown in Figure 3. The overall OR of HPV infection was 26.1 (95% CI 22.4 to 30.3). As far as genotypes were concerned, HPV type 73 had the highest OR, followed by HPV 16, 82, 45, and 51. The LR-HPV type with OR > 1 was HPV 11 and 42. Additionally, the prevalence of HR-HPV 83, LR-HPV 6, 43 and 44 in the abnormal specimens showed no significant difference to that in the normal specimens (Pearson’s Chi Square test, p > 0.05) and no ORs were supplied.

## Discussion

We presented a large clinic-based study (15,267 women) of HPV prevalence in the area of Zhejiang Province, Southeast China, from which interesting conclusions can be drawn. HPV detection and genotyping have been suggested to be incorporated in primary screening of cervical lesions [18],[19]. HPV typing is also very important for characterization of the population in HPV vaccination trials and for monitoring the efficacy of HPV vaccines [13]. Accordingly, determination of type-specific HPV prevalence in a region is considered to be one of the important steps towards cervical cancer prevention.

It was observed that the overall HPV infection was 22.8% in female outpatients in this study. The overall HPV prevalence reported from different regions of China varied greatly, ranging from 7.2% (Chaozhou, South China) to 36.5% (Harbin, Northeast China) [15],[20]. HPV infection in our study population was similar to that in Fujian, Southeast China (22.5%) [21], but higher than that in a previous population-based study in Zhejiang (13.3%) [22]. The difference might primarily be attributed to the fact that our work was a clinic-based study with a much larger study cohort. In addition to geographical differences, the observed HPV prevalence was also affected by the study cohorts (clinic-based or population-based), examination methods and time period [23].

We presented the age-related prevalence curves of HPV infections. The prevalence of total HPV, single HR-HPV and multiple HPV, all showed bimodal age distribution, with a peak at the ≤20 age group and a second one at the >60 age group. This has been observed in several other studies in China [1],[14],[24], but differs from most of the European countries, with a decrease in HPV prevalence after age 20 and a levelling off after age 45 [25],[26]. The actual first peak may due to the fact that younger women are more prone to have multiple partners [27] and are also less likely to have developed immunity to HPV [28]. The second peak of HPV infection in women aged >60 years should also arouse enough attention. The high infection rate around menopausal women may be attributed to viral persistence or reactivation of latent HPV caused by the physiologic and immunologic dysregulation at menopausal transition [29],[30]. Accordingly, HPV detection is clinically valuable for perimenopausal women in cervical cancer screening program. However, since HPV prophylactic vaccines do not have clinical benefit in women infected with vaccine types at the time of vaccination, we assume that younger women would obtain more benefit from “catch-up” HPV vaccination programs.

The five most common HPV types in women with normal cytology worldwide are HPV 16, 18, 31, 58 and 52, while the rank varied by region [31]. HPV 16 and HPV 18 are the most common oncogenic types associated with cancer, and are targeted by the vaccines [8],[12]. In our population, the five most common HPV types were HPV 16, 58, 52, 33 and 11 basing on the overall prevalence. Similarly to most previous surveys in China [15],[24] and other population [7],[32], HPV 16 was the most common type. Somewhat differently, HPV 58 and 52 were more prevalent than the vaccine type HPV 18 in our population. Moreover, no matter in normal, ASC-US, LSIL, ASC-H or HSIL cytology samples, HPV 16, 58 and 52 were always the major type, with the rank varied. Especially, HPV 58 was the most prevalent type in HSIL cytology samples. Some previous studies have shown that HPV 58 and 52 were more prevalent and overrepresented in cervical cancer cases in Asia [33],[34]. In China, it was observed that the two HPV types were more prevalent in the south and the southwest compared to other regions among women with precancerous lesions and cervical cancer [35]-[37]. The preponderance of HPV 58 and 52 in our study population is meaningful, which enhances the hypothesis that the second-generation HPV prophylactic vaccines including HPV 58 and 52 may offer higher protection for women in China and other Asian areas.

As far as cytology were concerned, it was observed that the overall HPV prevalence in normal, ASC-US, LSIL and HSIL cytology samples were 16.8%, 80.2%, 91.2% and 94.4%, respectively. Overall HPV prevalence increased as lesions progressed to higher grade ones, suggesting that HPV infection carries an increased risk of developing cervical neoplasia. The same trend was also observed in other regions or countries. For examples, in Henan, Central China, the overall HPV prevalence in normal, ASC-US, LSIL and HSIL cytology samples were 57.1%, 72.5%, 84.0% and 88.6%, respectively [38]. A population-based study of HPV genotype prevalence in America showed that the overall HPV infections were found in 24.3%, 57.9%, 94.6% and 95.5% of normal, ASC-US, LSIL and HSIL cytology samples, respectively [13]. The risk of each HPV type for cervical lesions was estimated using crude ORs. The overall OR of HPV infection was 26.1 (95% CI 22.4 to 30.3), showing that the cervical lesion risk of HPV-infected women increases to about 26-fold of those uninfected women. The five most risky HPV types were 73, 16, 82, 45 and 51. HPV 73 had the highest OR for abnormal cytology (ASC-US, LSIL, ASC-H and HSIL). Although the overall prevalence of HPV 73 was relatively low (0.2%), HPV 73 infection was at the highest risk of abnormality, which should be taken more attention to. The association of HPV 73 and HPV82 with cervical epithelial lesions were confirmed and the two types were classified as HR type in previous studies [6],[39]. HPV 73 was even more risky than HPV 16 based on HPV-type-specific risk analysis in Bahia, Brazil [40] and Northwest Germany [41], which was consistent with our results in this study.

## Conclusion

We presented a large cross-sectional study, from which baseline data of HPV prevalence in Zhejiang Province was provided. HPV prevalence showed bimodal age distribution, with a peak at the ≤20 age group and a second one at >60 age group. HPV 16, 58 and 52 were the most commonly identified HR-HPV types while the vaccine type HPV 18 showed relatively low prevalence. As expected, HPV prevalence increased as lesions progressed to higher grade ones. HPV 73, 16, 82, 45, and 51 were the most risky types in this area. Our data will provide guidance for the primary screening and vaccination program for cervical cancer.

## Authors’ original submitted files for images

Below are the links to the authors’ original submitted files for images. Authors’ original file for figure 1 Authors’ original file for figure 2 Authors’ original file for figure 3

## Abbreviations

HPV: Human papillomavirus
LR: Low-risk
HR: High-risk
ASC: Atypical squamous cell
ASC-US: Atypical squamous cells of undetermined significance
ASC-H: Atypical squamous cells that cannot exclude HSIL
SIL: Squamous intraepithelial lesion
LSIL: Low-grade SIL
HSIL: High-grade SIL
ORs: Odds ratios
CI: Confidence interval
TCT: ThinPrep cytology test
