# Supplementary figures and images for: Human papillomavirus prevalence and type-distribution among women in Zhejiang Province, Southeast China: a cross-sectional study
Source: BMC Infect Dis. 2014 Dec 19;14:708. doi: 10.1186/s12879-014-0708-8 (PMC4278233; doi:10.1186/s12879-014-0708-8)

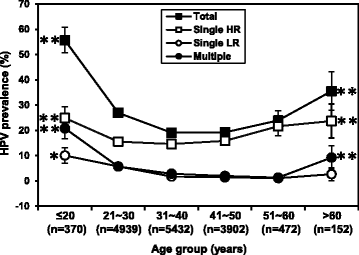

Supplement: Supplementary file 1 — Authors’ original file for figure 1 [file 12879_2014_708_MOESM1_ESM.gif]

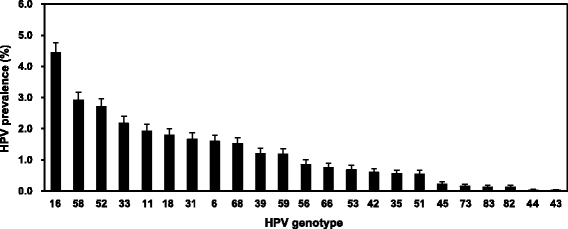

Supplement: Supplementary file 2 — Authors’ original file for figure 2 [file 12879_2014_708_MOESM2_ESM.gif]

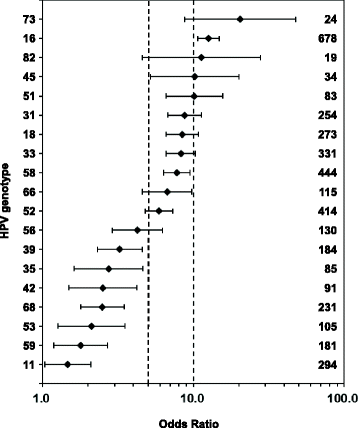

Supplement: Supplementary file 3 — Authors’ original file for figure 3 [file 12879_2014_708_MOESM3_ESM.gif]
